# Diatom Milking: A Review and New Approaches

**DOI:** 10.3390/md13052629

**Published:** 2015-04-29

**Authors:** Vandana Vinayak, Kalina M. Manoylov, Hélène Gateau, Vincent Blanckaert, Josiane Hérault, Gaëlle Pencréac’h, Justine Marchand, Richard Gordon, Benoît Schoefs

**Affiliations:** 1 Department of Criminology & Forensic Science, School of Applied Sciences, Dr. H.S. Gour University (Central University), Sagar Madhya Pradesh, India; E-Mail: kapilvinayak@gmail.com; 2 Department of Biological & Environmental Sciences, Georgia College and State University, Milledgeville, GA 31061, USA; E-Mail: kalina.manoylov@gcsu.edu; 3 MicroMar, Mer Molécules Santé, IUML—FR 3473 CNRS, University of Le Mans, Faculté des Sciences et Techniques, Avenue Olivier Messiaen, 72085 Le Mans cedex 9, France; E-Mails: lngateau@gmail.com (H.G.); justine.marchand@univ-lemans.fr (J.M.); 4 MicroMar, Mer Molécules Santé, IUML—FR 3473 CNRS, University of Le Mans, IUT de Laval, Rue des Drs Calmette et Guerin, 53020 Laval Cedex 9, France; E-Mail: vincent.blanckaert@univ-lemans.fr; 5 ChimiMar, Mer Molécules Santé, IUML—FR 3473 CNRS, University of Le Mans, IUT de Laval, Rue des Drs Calmette et Guerin, 53020 Laval Cedex 9, France; E-Mails: gaelle.pencreach@univ-lemans.fr (G.P.); josiane.herault@univ-lemans.fr (J.H.); 6 Gulf Specimen Aquarium & Marine Laboratory, Panacea, FL 32346, USA; 7 C.S. Mott Center for Human Growth and Development, Department of Obstetrics & Gynecology, Wayne State University, 275 E. Hancock, Detroit, MI 48201, USA; E-Mail: DickGordonCan@gmail.com

**Keywords:** diatom, biotechnology, milking, physiology, stress, biofuel, secondary metabolites

## Abstract

The rise of human populations and the growth of cities contribute to the depletion of natural resources, increase their cost, and create potential climatic changes. To overcome difficulties in supplying populations and reducing the resource cost, a search for alternative pharmaceutical, nanotechnology, and energy sources has begun. Among the alternative sources, microalgae are the most promising because they use carbon dioxide (CO_2_) to produce biomass and/or valuable compounds. Once produced, the biomass is ordinarily harvested and processed (downstream program). Drying, grinding, and extraction steps are destructive to the microalgal biomass that then needs to be renewed. The extraction and purification processes generate organic wastes and require substantial energy inputs. Altogether, it is urgent to develop alternative downstream processes. Among the possibilities, milking invokes the concept that the extraction should not kill the algal cells. Therefore, it does not require growing the algae anew. In this review, we discuss research on milking of diatoms. The main themes are (a) development of alternative methods to extract and harvest high added value compounds; (b) design of photobioreactors; (c) biodiversity and (d) stress physiology, illustrated with original results dealing with oleaginous diatoms.

## 1. Introduction

The rise of human populations and the expansion of cities into the countryside contribute to an accelerated depletion of natural resources [1], thereby increasing the prices of these resources in commercial markets and potentially changing the Earth’s climate. To overcome difficulties in supplying populations and reducing the resource cost, an intensive search for alternative pharmaceutical, food, high value molecules (HVM), and energy sources, including via genetic engineering and nanotechnology, has started [2,3,4,5,6,7,8,9,10]. Among the alternative sources, microalgae are promising because using their efficient capacity to photosynthesize, microalgae convert light energy into chemical energy into organic molecules such as carbohydrates and lipids. These molecules are synthesized using the carbon dioxide (CO_2_) from the environment. Among algae, diatoms (Bacillariophyceae) are responsible for a large part (up to 41%–50%) of the CO_2_ fixed in oceans [11,12]. In some circumstances, microalgae synthesize secondary metabolites [2,10,13,14,15] that can have important applications for food, health, cosmetic, energy or pharmaceutical industries. These compounds are called high value molecules (HVM). For instance, the price of the natural carotenoid astaxanthin is expected to reach $14,000 US kg^−1^ in 2018 [16]. Due to the diversified evolutionary history, the chemical diversity of HVM synthesized by microalgae is broad. Table 1 presents the main storage HVM, including oil content, of some microalgae.

Nowadays, algae are important biotechnological tools with applications in various industrial fields [17,18,19]. Examples are:

1. wastewater treatment [20,21,22,23,24],
2. biofuel production [24,25,26,27],
3. manufacture of fertilizers [28],
4. production of secondary metabolites for pharmaceutical products [2,29,30],
5. production of food for humans [31,32,33],
6. animal feed [34,35,36], and
7. medical compounds [2,37,38,39].

**Table 1:** Main storage compounds and oil percentage of some microalgae.

Phylum	Class	Taxonomy	Oil Content (% d.w.)	High Value Molecules	Reference
Chlorophyta	Chlorodendrophyceae	*Tetraselmis suecica*	15–32	Carotenoids, chlorophyll, tocopherol, lipids	[40]
Chlorophyta	Chlorophyceae	*Ankistrodesmus* sp.	28–40	Mycosporine-like amino acids, polysaccharides	[17]
Chlorophyta	Chlorophyceae	*Dunaliella salina*	10	Carotenoid, β carotene, mycosporine-like amino acids, sporopollenin	[41]
Chlorophyta	Chlorophyceae	*Dunaliella tertiolecta*	36–42	Carotenoid, β carotene, mycosporine-like amino acids	[42]
Chlorophyta	Chlorophyceae	*Neochloris oleoabundans*	35–65	Fatty acids, starch	[43]
Chlorophyta	Trebouxiophyceae	*Botryococcus braunii*	29–75	Isobotryococcene, botryococcene, triterpenes	[44]
Chlorophyta	Trebouxiophyceae	*Chlorella vulgaris*	58	Neutral lipids	[45]
Chlorophyta	Trebouxiophyceae	*Chlorella emersonii*	34	Neutral lipids	[46]
Chlorophyta	Trebouxiophyceae	*Chlorella protothecoides*	15–55	Eicosapentaenoic acid (EPA), ascorbic acid	[47]
Chlorophyta	Trebouxiophyceae	*Chlorella minutissima*	57	C16- and C18-lipids	[48]
Heterokontophyta	Bacillariophyceae	*Nitzschia laevi*	28–69	EPA	[49]
Heterokontophyta	Coscinodiscophyceae	*Thalassiosira pseudonana*	21–31	Glycosylglycerides, neutral lipids, TAG	[50]
Heterokontophyta	Labrynthulomycetes	*Schizochytrium limacinum*	50–77	Docosahexaenoic acid (DHA)	[51]
Myzozoa	Peridinea	*Crypthecodinium cohnii*	20	DHA, Starch	[52]
Ochrophyta	Coscinodiscophyceae	*Cyclotella* sp.	42	Neutral lipids	[53]
Ochrophyta	Eustigmatophyceae	*Nannochloropsis* sp.	46–68	EPA, TAG, ω-3 LC-PUFA	[54]

Another reason to be interested in microalga-based biotechnological processes is that they are expected to be more productive per unit surface area of Earth than any cultivated agricultural plant [55,56]. For instance, diatom oil production is predicted to be 6–200 times higher than oilseed crops per unit surface area [27,57,58].

Although the use of microalgae for the production of HVM is very recent, microalgae played a crucial role in the formation of crude oil deposits in ocean floors, which are a rich natural source of fossil fuel [59]. This contribution continues today because algae are responsible for a large fraction of the organic carbon being buried on continental margins [60] and oceanic diatoms may even alleviate predicted global warming [61].

In current processes, the algal biomass is harvested, water is removed and HVM are extracted and purified. Milking processes aim to keep the algae alive while HVM are extracted, allowing indefinite repetition of the process. The milking approach will be more environmentally and economically friendly, if it comes to commercial fruition.

**Figure 1:**
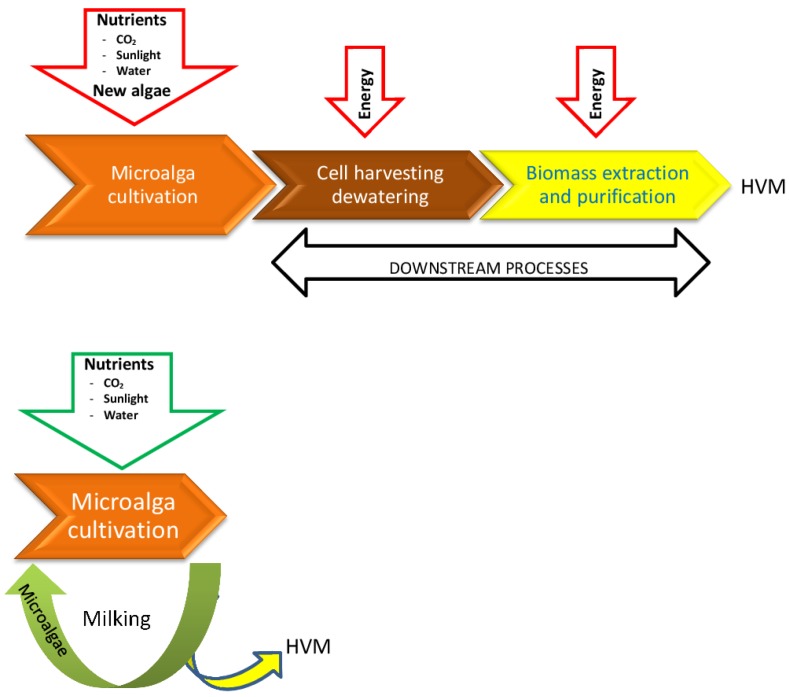
Scheme presenting milking as an alternative to current processes. (**Top**): Current processes are systems that require starting new microalgal cultures for each batch of HVM, with fresh nutrients; (**Bottom**): Milking only requires inputs of carbon dioxide and water used up in producing HVM, and thus is closer to a closed system.

Whatever the biotechnological process used and the final products obtained from the algae, they all start with growing algae to obtain biomass. It is usually considered that microalga growth presents low productivity both in terms of biomass and product formation [62]. Therefore, a first critical issue in developing alga biotechnology is the optimization of culture parameters [63,64,65] (Figure 1). Once produced, the biomass is harvested and processed according to a dedicated downstream program generally involving dewatering, drying, grinding, extraction, and purification steps [65]. Grinding [66] and extraction [67] are the most popular steps for separation of the HVM from the organisms [65,68] (Figure 1). Obviously, drying and grinding require energy inputs and kill the microalgal biomass that then needs to be repeatedly regrown. In addition, extraction and purification processes generate organic wastes. A critical assessment of current microalga bioprocessing technology reveals that downstream processing also poses a number of important technical and economic challenges ([65,69,70] and references therein).

“No harvesting method has yet been identified as being efficient, reliable and at reasonable cost”.

*[71]*

For instance, it is estimated that downstream operations costs usually represent 50%–80% of the total processing costs [72,73,74,75,76,77,78]. Moreover, some of the currently proposed downstream processes are not economically sustainable, because they depend on waste CO_2_ [79,80,81,82,83], which is a nonstarter for large-scale production. This can be seen as follows. In 2011, 21% of CO_2_ emissions were industrial, while 22% were due to transport, and the latter figure is projected to reach 40% by 2035 [84]. This means that even with 100% utilization of industrial CO_2_ waste, there is not enough to be converted to biofuel for all transport now, let alone by 2035. So although the use of atmospheric CO_2_ may slow HVM production [85], the use of waste CO_2_ is at best a stopgap.

Altogether, it seems urgent to develop alternative downstream processes. Among the possibilities, the concept of milking was introduced by Frenz and collaborators in 1989 [86] to describe the extraction of hydrocarbons from the green algae *Botryococcus braunii* Kützing (Trebouxiophyceae) by exposing the cells for a short time to hexane (cf. [87]). Ramachandra *et al*. [27] have pointed out the analogy to grinding up cows to extract dairy milk, which has been acknowledged, but regarded as inevitable:

“Plenty of organisms use those same inputs—All photosynthetic microalgae, for example. But you can’t milk them like a cow. You have to crush them”.

*[79]*

The milking concept specifies that the extraction should not kill the cells. Consequently, milking does not require the constant need of culturing and re-growing the entire stock of algae, which has a typical timescale of a few hours to weeks. If only hydrocarbon HVM were milked, then the need for nitrogen/phosphorus fertilizers would be reduced [88] or eliminated.

In some of the literature the distinction that we are now making between extraction and milking was not considered, so that what we are calling extraction was labeled as “milking” [67]. Similarly, we previously lumped secretion and extraction under “milking” [27,89]. We think that careful distinctions between these approaches will make for better dialogue. Thus we define:

- Milking as the removal of a specific set of products without killing the organism, in such a way that it can be milked again at a later time.
- Extraction as the removal of a specific set of products without concern about the survival of the organism, generally leaving an organic residue.
- Secretion or spontaneous oozing as the active dumping of a specific set of products by an organism into its surrounding environment.

In this manuscript, we propose food for thought toward the development of milking of microalgae, particularly diatoms. To reach our goal, the main grounds for thought that we have identified are (a) development of alternative methods to extract and harvest HMV; (b) design and functioning of photobioreactors; (c) biochemodiversity and (d) diatom (stress) physiology. They are discussed separately in this contribution and, when possible, illustrated with original results dealing with diatoms accumulating lipids. These algae are referred to as oleaginous diatoms. Although recent reviews [90,91] addressed several points discussed in this contribution, none of them focused on diatoms and milking.

## 2. Development of Alternative Processes to Extract and Harvest High Value Molecules (HVM)

The key element of milking is finding a way to extract HVM without killing the HVM-producing cells. This concept was first applied to higher plants by milking, for instance, rubber 2000 years ago [92]; maple syrup historically [93] and prehistorically [94,95]; turpentine as long ago as Hippocrates [96]; and, more recently, halophilic bacteria [97,98] and microalgae. The first applications with algae used biocompatible solvents to extract HVM (reviewed in [27,62]). Recently, Gillet [99] calculated the molecular dynamics of the plasma membrane during milking using organic solvents. The conclusion of this theoretical study agreed with earlier experimental results obtained by Zhang *et al*. [67] and showed that hexadecane is the best organic solvent to milk lipids from *Nannochloropsis* sp. (Eustigmatophyceae) (*cf.* [100]). However, solvents alone are usually not efficient for HVM extraction if the cell wall is too robust [101]. In this case, HVM extraction should be assisted by other methods. It is important to recall here that the efficiency of these processes may depend on the growth stage of the cells. Table 2 presents an overview of these different potential technologies with a summary of their strengths and weaknesses, although without any pretention of being exhaustive. The aim of this section is to provide a detailed analysis of these possibilities in the framework of a milking strategy. We do not consider destructive processes, such as microwaving and heating [102], though in moderation they might also serve to induce milking.

**Table 2:** Extraction processes potentially applicable for milking of microorganisms.

Milking Process	Microorganism	Advantages	Disadvantages	Ability to Keep Cells Alive
**Biocompatible organic solvents**	Microalgae [67,100]	Improvement of lipid production Positive effect on growth	Not environmentally friendly Possible toxic mechanism	Yes, when using hydrophobic solvents
**Pulsed electric field (PEF)**	Yeast [103] Microalgae [104,105,106,107] Cyanobacteria [108]	High extraction yield Adjustable PEF parameters Not an energy-intensive process Large-scale process demonstrated Continuous process	Effect of electric pulsation is size dependent	Yes, but depends on the PEF parameters
**Spontaneous oozing**	Microalgae [109] Bacteria [110,111,112] Cyanobacteria [113,114,115]	Not an energy-intensive process Possibility of scaling up Application in solar panels	Slow oozing of HVM	Yes, it is a natural mechanism
**Mechanical methods**				
-sonication	Microalgae [116,117] Cyanobacteria [118,119,120]	Improvement of lipid recovery	Cellular damage apoptosis Thickness of the cell wall	No
-pressure	Microalgae [this work]	Not an energy-intensive process Weak pressure to be used (below 750 µN)	Large-scale process not demonstrated Process needs to be improved	Yeswhen using low pressure (< 750 µN)
-centrifugation	Diatoms [work in progress]	Continuous process Application in solar panels	Requires energy	Not yet tested
**Membrane-bound protein pumps**	Bacteria [121,122]	Oozing of HVM Lower toxicity of overexpressed HVM High rate growth Possibility of scaling up	Metabolism engineering Organic phase needed for solubilization of water insoluble HVM	Yes

### 2.1. Pulsed Electric Field

Electroporation has been applied to diatoms and other microalgae, albeit primarily to allow genetic transformation [123,124,125,126,127,128]. However, the application of trains of electric pulses can also be used to favor the release of HVM from cells. In this method, cell membranes are punctured by electric pulses, subsequently allowing the cell components to ooze out. The method was first established with yeast [103,129] and more recently applied to photosynthetic organisms such as cyanobacteria [108] and microalgae [104,105]. The “punctured” cells then heal and remain viable and the same batch of algae can be reused for further extraction of HVM [106]. Because electric pulses can have deleterious effects in bacteria [130], algae [131] and mammal cells [132], such as irreversible electroporation, the choice of the characteristics of the electric pulses such as length, intensity, polarity, frequency of repetition, *etc*. require a compromise between milking efficiency and algal survival. The tuning of physical parameters is of importance because the effect of the pulsed electric field is cell size dependent: the smaller the cell, the stronger the electric treatment [105,133,134]. Repetitive 2 ms long electric pulses of alternative polarities with field strength of 3 and 6 kV/cm were capable of significantly inducing the extraction of cytoplasmic proteins from two freshwater microalgae, *Chlorella vulgaris* Beyerinck (Trebouxiophyceae) and *Haematococcus pluvialis* Flotow (Chlorophyceae), both with rigid cell walls [105]. A post-pulse incubation step was found to be necessary in order to allow oozing, but also to permit the algae to recover between trains of electric pulses [105,107,135].

### 2.2. Spontaneous Oozing

**Figure 2:**
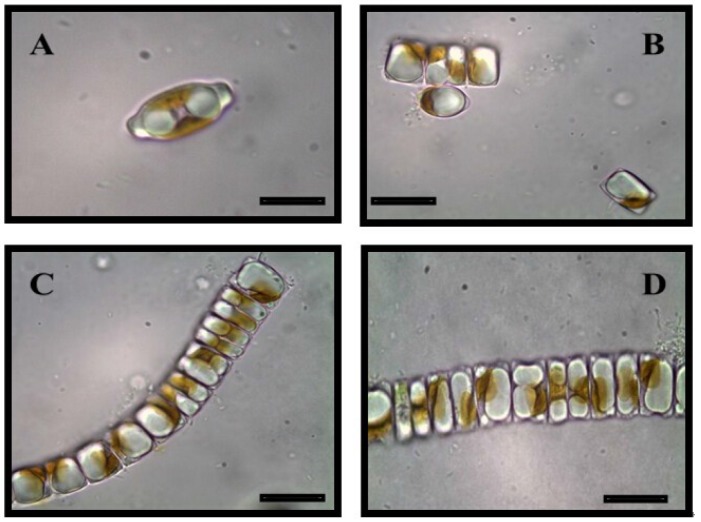
*Diadesmis confervaceae* in solitary and chain forms as observed under 100× oil immersion. Note oozed oil droplets in panel C. Cf. [109]. Scale bar: 10 µm.

Currently, there are proprietary reports that some bacteria [110,111,112], green algae [62,86] and genetically engineered cyanobacteria [113,114,115,136,137] can secrete lipids [82] from their cytoplasm to the external environment. *Botryococcus* secretes its oil into a kind of cell wall called the outer matrix [87]. Surface properties may prove important in secretion [138]. Very recently, Vinayak *et al.* [109] reported that a small diatom strain (14–18 µm long and 6–7 µm width), *Diadesmis confervaceae* Kützing (Figure 2), producing 14.6% lipid content, exudes lipid droplets into the culture medium. The mechanisms involved in oozing are not yet determined. The droplets accumulate either in the chloroplasts (plastoglobules) or/and in the cytoplasm (oleosomes) (see also Section 4).

### 2.3. Mechanical Pressure

In algae lacking a natural oozing mechanism, one could assume that exerting a mechanical pressure, such as ultrasound or touch, could force HVM to come out of the cells. Similarly to electric pulse treatment (Section 2.1), ultrasound has been used in processes for improving the extraction of carotenoids (*Haematococcus pluvialis* [139,140]), chlorophyll (*Chlorella* sp. [141], *Dunaliella* [142,143]), and lipid (*Chlorella vulgaris* [116]) (for a review, see [144]). Because ultrasound effects can be harmful (death [118], nonviable cellular damage [119], or induction of programmed cell death [120]), the characteristics of the ultrasound treatment should be chosen to keep the cells alive and, thus, be qualified for inclusion in a milking process. For instance, Araujo *et al.* [116] reported that ultrasound treatment of *Chlorella vulgaris* produced a significant improvement in lipid recovery when compared to the yield reached in the absence of the treatment. However, the efficiency of the treatment depends on the cell wall strength as only a weak improvement was recorded with *Scenedesmus*, a Chlorophyceae taxon with tougher cell walls [117,118]. For instance a 20 min, 20 kHz treatment causes death of the cyanobacterium *Microcystis aeruginosa* Kützing [118].

**Figure 3:**
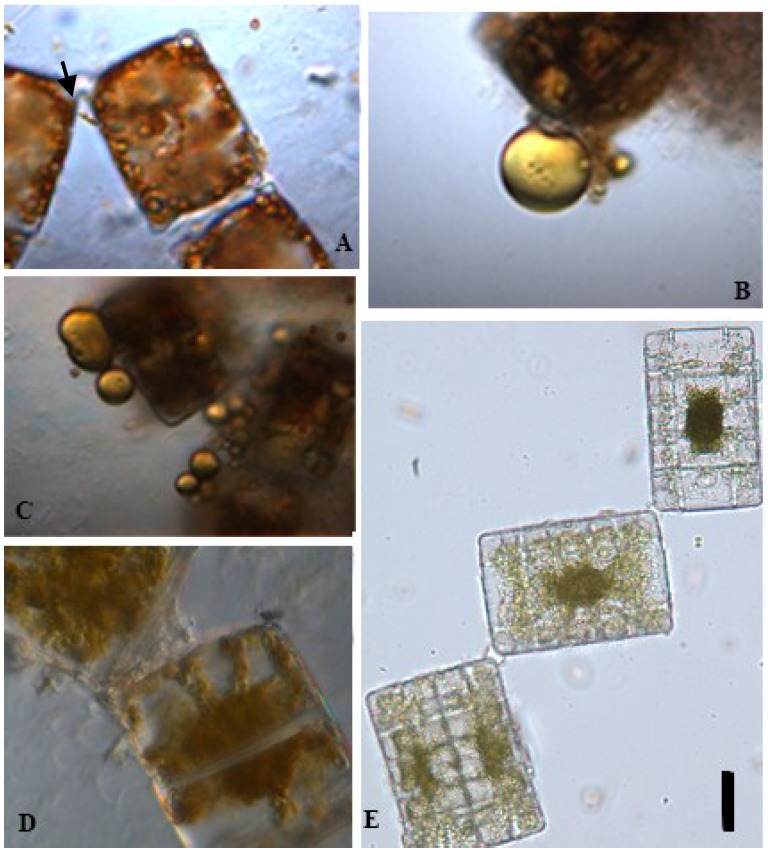
*Terpsinoë musica* is a freshwater diatom that releases oil under mechanical pressure on the cover slip. Scale bar = 10 µm. (**A**) Freshly collected *Terpsinoë musica*; (**B**,**C**) *Terpsinoë musica* cells kept 7 days in an incubator released oil when mechanically pressed; (**D**) *Terpsinoë musica* divides 10 days after oil release; (**E**) Zigzag colony of *Terpsinoë musica*.

Diatom cells present the unique feature of being enclosed in a hydrated silicon dioxide cellwall denoted the frustule. Frustule shape and decorations are widely diversified [145,146]. In addition, each diatom has an imperfect bilateral symmetry since one of the frustules is slightly larger than the other, allowing one valve to fit inside the edge of the other. Because of this and the robustness of the frustule, mechanical methods could provide a very powerful method favoring the release of HVM. To test this possibility, diatoms harvested in Georgia, USA (see Section 3) were placed in water on a rigid surface and covered with an 18×18 mm^2^ coverslip and pressed with a microbial needle. Pressure was applied in the middle of the coverslip until water flow out from under the coverslip ceased. All expressed water remained on the slide at the edges of the coverslip. The population remaining under the coverslip was then visually scanned and changes such as breaking of the cells or release of oil were documented. Where possible to identify them, the same cells were scrutinized. With the mechanical pressure on the cover slip live diatoms were not visibly broken or harmed and the release of oil from *Terpsinoë musica* Ehrenberg was recorded at least twice. Interestingly, *Terpsinoë musica* naturally produce zigzag colonies (Figure 3, panel A,E) in which the individual cells are attached by organic pads secreted from the terminal apical pore fields (Figure 3A arrow and 3B) [147]. The observations reported here suggest that the oil is also released through the apical pore field, which is also known to be used for the release of carbohydrates [148]. It would be interesting to test other diatoms with an apical pore field [149] for their oozing capacity. Although the force that has to be exerted on a live diatom for expressing oil has not yet been measured, we can assume that it is considerably less than the force required to break an isolated diatom valve. Hamm *et al.* [150] presented mechanical properties of various diatoms under tensile and compressive stresses, and reported the breaking force for an isolated diatom valve to be 750 μN.

### 2.4. Centrifugation

At the time of writing, we have just started work on release of oil from diatoms via centrifugation, which will be reported later. We have yet to determine if the centrifugal force that releases oil is lower than that which kills them. If so, the centrifugation could become a milking technique. Centrifugation is presently used for algal separation [151]. In sea urchin eggs centrifuged at 9000*× g* the lipids rise to the centripetal end and exert enough buoyancy to divide the egg in two [152].

## 3. The Design and Functioning of Photobioreactors

In microalgal biotechnology, the cultivation of biological material is considered as one of the major constraints to commercial development, despite the fact that maximum doubling rates between 2 and 5 day^−1^ have been reported [27,153]. The simplest method for algae production is the open raceway pond: shallow, circulating, open bodies of water that are exposed to the elements and use the natural energy of the sun [154,155]. There are several advantages to this design, such as less material and capital being required for operation, but there are many drawbacks [65,156,157,158]. Primarily, the open nature of a raceway pond leaves it vulnerable to contamination, low volumetric productivity and poor temperature and light intensity control [159]. Photobioreactors (PBRs) allow much more control than open raceway ponds due to their closed systems [73,74,160,161]. They are usually made of glass or plastic and can be used outside with natural sunlight as well as indoors with artificial light such as light-emitting diodes (LEDs) [162,163]. The three main designs, annular PBRs, which are large columns, flat panel PBRs, and tubular PBRs, allow for laboratory conditions at all times. Yet, this approach is considerably more expensive than the open raceway pond [65,76,164,165]. As far as oil production is concerned, for all methods to be economically viable, they either have to be cheaper than traditional gasoline and be seamlessly introduced into an infrastructure based on fossil fuels, or they need to be protected by tariffs or embargos on imported oil [166] and/or subsidized relative to fossil oil. To involve the milking philosophy into microalgal biotechnology, downstream processes have to be modified to allow milking operations such as electric pulses to occur.

One can also propose innovative photobioreactors such as the live diatom solar panel [27]. Such a panel may require sandwiching diatoms in a series of polymeric microchannels that can be squeezed by increasing pressure on the backside of the channel. Forces of this magnitude can be produced in a microfluidic environment by electrical and mechanical means. The former mechanism can utilize Maxwell stresses induced by specifically designed electrode systems [167,168]. One suitable material for such polymeric channels is polydimethylsiloxane (PDMS), which can covalently bond to glass during the solar panel manufacturing process, and can be prepared with a long-lasting hydrophilic surface suitable for cell culture [169]. PDMS is impermeable to water but would transmit air including much needed CO_2_ [170] to diatoms. The front of the glass-PDMS chambers can provide a fluidic environment to diatoms, while the backside of the PDMS chambers can be interfaced with gas channels. Increasing pressure in the gas channels would push the flexible PDMS and squeeze diatoms between the PDMS and glass substrates, possibly releasing oil. Large-scale systems with thousands of individually controlled PDMS micro-valves have been demonstrated before [171]. The diatom solar panel envisioned would require only several valves and the entire system could be fabricated cheaply, making this proposed technology cost effective. Separation of milked oil from the diatoms could perhaps be facilitated by buoyancy, pumping, centrifugation and/or the use of spatial gradients or step functions of the surface hydrophobicity of the PDMS channels [172]. If the diatoms used in diatom biofuel solar panels do not spontaneously secrete HVM, then a power source to apply force to the live diatoms to milk them for biofuel (see Section 2) would be needed (possibly using solar electric power generated in the same panel). Instead of bulk solvent extraction, the algal cells could be left in place and, lipids and/or other HVM removed by microfluidics [173].

In this review our focus is on the use of living diatoms to produce biofuel and other compounds. It is important to distinguish this from the use of nonliving diatom frustules in novel electricity-generating solar panels [174]. Of course, both could be combined in a single panel.

Solar panels containing living diatoms or other algae could go on rooftops, for example, and we have estimated that surface areas of 10 square meters per person might suffice to match USA rates of gasoline consumption [175], especially if the algae could be selectively bred [176] or genetically engineered for octane production [177] or other low carbon and volatile compounds [27]. Diatom solar panels also have the advantage of local gasoline production, reducing the need for a distribution system, storage of energy overnight in liquid form instead of in heavy batteries that last only a few years [178] and could make use of species adapted to local conditions [179]. While developing new processes, especially algal solar panels [27,180], one should keep in mind that algae that are in use must stay alive. Species should therefore be identified as, for instance, from geothermal waters that are thermo-resistant (as are some green microalgae [181]) or thermophilic [27] (as some diatoms are [182,183,184,185]).

## 4. Diatom Chemobiodiversity

Tens of thousands of strains of microalgae have been described, millions of species are expected to exist [186,187], and new species, including diatoms, are continuously being described. In contrast to this amazing biodiversity, no more than 10 species are commonly used at the commercial level, regardless of the geographical location of the producing company, which is often done outdoors [65,188]! These species include the diatoms *Phaeodactylum tricornutum* and *Odontella aurita* [16]. Thus another way forward is the development of more strains of industrial interest [173]. This is especially important if HVM production units are widely distributed from the geographic point of view as prospective studies have predicted [189]. In other words, promising strains must be optimized for local climatic conditions, and media optimized for those strains [190]. Bloom diatoms, for example, may be particularly good oil sources [191], and perhaps invasive diatoms [192] would be worth considering. There are several nonexclusive possibilities for finding such species/strains: (a) strain selection, (b) synthetic biology and (c) exploring biodiversity. Study of biodiversity can also lead to the discovery of new HVM.

### 4.1. Strain Selection

In the introductory paragraph of this section, we underlined how it will be quite important to use dedicated species for the production of HVM. Because methods for synthetic biology are still under development for microalgae (see Section 4.3), screening remains one of the most promising methods for the selection of dedicated species provided a selection criterion has been determined and a fast and low cost detection method is available [193]. For instance, Kopecky *et al.* [194] screened algal collections for the capacity of green algae to synthesize secondary carotenoids. Using high light stress as a selection pressure and thin-layer chromatography as the analytical method several putative, interesting strains were characterized for their secondary pigment content. Recently, nondestructive methods such as Fast-Fourier InfraRed (FT-IR) spectroscopy have been developed to characterize the biochemical contents of microalgae [195,196,197].

The selection pressure need not be a natural factor. Bougaran *et al.* [198] developed a nongenetically modified organism mutation-selection method based on UVC irradiation (at 254 nm wavelength) followed by flow cytometry selection to isolate an *Isochrysis affinis galbana* population overaccumulating neutral lipid (with a productivity increase of 80%). Robert *et al.* [199] used the herbicide metolachlor, a member of the chloroacetamide family of compounds, to select diatom strains with hyperaccumulation of long-chain polyunsaturated fatty acids (PUFA). PUFA are health-benefitting molecules [2,200]. This herbicide was chosen because it is an inhibitor of the fatty acid ester-type (FAE) elongase biosynthesis of very long-chain fatty acids in microalgae [201]. For a review on the metabolic pathways leading to very long chain fatty acids, see [14].

### 4.2. Exploring Biodiversity

It is usually admitted that biodiversity constitutes the richest source of bioactive molecules [202]. Unfortunately, since World War II, the search for such compounds from natural sources has been gradually replaced by synthetic and combinatorial chemistry (for a review, see [203]). In the recent past, many different scale bioprospecting programs such as the Aquatic Species Program [204] or Tara Oceans Cruise [205] have aimed to find new HVM from nature [206,207,208]. The Aquatic Species Program identified 50 promising microalgal strains for biofuel production among which 60% are diatoms. This number is far greater than the number of diatom species that have been used so far in microalga biotech. This shift back to bioprospecting programs partly relies on the assumption that living organisms are a storehouse for HVM that are just waiting to be discovered. Theoretical investigations of the relationship between biological and chemical diversities revealed that it is unlikely that a fine combing of global resources sharing similar biological niches will discover interesting species unless they are distant from the phylogenetic point of view, as shown for the plant–herbivore relationship [209]. Therefore, bioprospecting would be more successful in screening biological resources, including microalga collections, from diversified ecological niches such as hot sources [181,185], depths of the ocean [181,210], or very saline or polluted waters [145] with a search for organisms that are phylogenetically distant from one another [211]. Once new strains have been collected from the natural environment, they should be selected according to defined criteria through a screening program as explained in Section 4.1 (phytohormones [212], lipids [213]). For instance, various places in Georgia were sampled for diatoms from March to July 2011 in triplicate (Table 3).

**Table 3:** Site locality and identification for sites collection within southeastern USA, temperature (T), pH, percent dissolved oxygen (DO %) and conductivity (µS cm^−1^), mean ± SE.

Location	No.	T (°C)	pH	DO %	Conductivity	Latitude	Longitude
Lake Sinclair Power Plant	1	23.5 ± 4.2	7.0 ± 0.2	9 ± 2.52	32 ± 3.6	33.20	−83.30
Lake Sinclair, Goat Island	2	21.8 ± 1.3	8.5 ± 0.1	110 ± 14.1	67 ± 6.2	33.16	−83.23
Lake Sinclair at Dam	3	19.9 ± 6.1	7.8 ± 0.8	69 ± 8.2	46 ± 7	33.14	−83.20
Oconee River at Dam	4	23.8±5.1	7.1± 2.1	61 ± 11.1	82.6 ± 1.4	33.14	−83.20
Oconee River Greenway	5	20.2 ± 9	7.2 ± 1.6	75 ± 2.6	78.3 ± 6.9	33.08	−83.21
Fishing Creek	6	22.4 ± 3.2	6.3 ± 0.4	89 ± 8.2	29.2 ± 11.4	33.08	−83.22
Tobler Creek	7	22.1 ± 2.1	7.2 ± 0.2	45 ± 10.6	85 ± 5.3	33.12	−83.27
Andalusia pond	8	23.5 ± 1.8	6.8 ± 0.5	76 ± 9.6	24.3 ± 1.8	33.13	−83.27
Bartram forest pond	9	19.5 ± 3.2	7.4 ± 1.3	58 ± 12.6	78.5 ± 1.6	33.02	−83.21
Savannah River at Port Wentworth, GA	10	25.5 ± 4.4	7.6 ± 0.4	86 ± 9.7	8204 ± 125.4	32.17	−81.16

Five lentic (three locations at Lake Sinclair, ponds at Andalusia Farm and Bartram Forest, Baldwin County) and five lotic locations were sampled; two on Oconee River and one each at Savannah River, Tobler and Fishing Creeks, in southeastern Georgia, USA (Table 3), all locations being freshwater with the exception of the samples from the Savanna River at Port Wentworth, GA with conductivity higher than 2000 µS cm^−1^. Each habitat sample was taken as composite vegetation; water and sediment samples were taken following the loose sediment collection technique in EPA’s periphyton protocols [214]. Representatives of 24 diatom genera were identified from the natural samples with 314 taxa identified to species from all samples. The brackish sample from the Savannah River estuary was the most diverse with 167 diatom species. The freshwater diatom community was dominated by representatives of pennate genera including *Nitzschia*, *Fragilariforma*, *Synedra* and *Eunotia* (see Supplementary Table S1).

**Figure 4:**
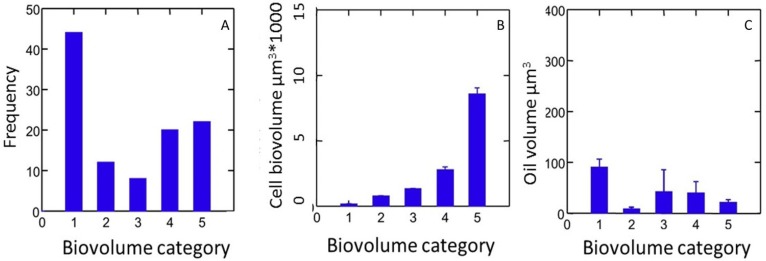
Frequency of cells in five diatom cell biovolume categories, for cells that contained lipid droplets. (**A**) Frequency of cells in 5 diatom cell biovolume categories, for cells that contained lipid droplets; (**B**) Average biovolume within each cell biovolume category; (**C**) Oil content within each cell biovolume category.

Cell size and the relationship between size and oil production were tested with the data. Only 106 individual cells out of thousands visually scanned (representing 341 species) had visible lipid droplets. Small diatoms dominated the size distribution of those diatoms containing lipid droplets and the frequency numbers above the highest potential average of oil content within that category (Figure 4). Only representatives of the genera *Craticula*, *Pinnularia*, and *Eunotia* from natural freshwater conditions and *Pleurosira* and *Terpsinoë* from the brackish environment had visible lipid droplets. None of the araphid diatoms observed had visible lipid droplets. Some pennate diatoms such as *Pinnularia* and *Craticula* representatives were shown to have less than 10% volume concentrations of oil within the first 7 days (see also Supplementary Data S2).

### 4.3. Toward Dedicated Producers Using Synthetic Biology

Despite the promises of studying chemobiodiversity for industrial processes, the chance of finding, in a short time, perfect strain(s) adapted to the *ad hoc* industrial environment, growing quickly and producing high amounts of specific compounds is as difficult as finding a needle in a haystack. This reasoning led to the concept of engineered cells as HVM factories. In this concept, such cells would be tailored to be capable of producing dedicated HVM and could be used to tackle some of society’s most important and/or toughest challenges [215].

Diatoms have been successfully transformed by microprojectile bombardment (*Chaetoceros* sp. [216], *Cyclotella cryptica* [217], *Cylindrotheca fusiformis* [218,219], *Fistulifera* sp. [220], *Navicula saprophila* [217], *Phaeodactylum tricornutum* [221,222], *Thalassiosira pseudonana* [219]. Unfortunately, microprojectile bombardment is costly and does not allow a high transformation efficiency. To overcome these difficulties, electroporation transformation protocols have been developed for *Phaeodactylum tricornutum* [126,128,223] and *Chaetoceros gracilis* [224]. They allow a maximum transformation efficiency (2.8 × 10^−5^ cells) comparable to the ones obtained with *Chlamydomonas* sp. (2.0 × 10^−5^ cells [225]) or *Nannochloropsis* sp. (1–2 × 10^−5^ cells [226,227]).

Several publications established the benefit of genetic engineering for enhancing the production of HVM [228,229]. For instance, Trentacoste *et al*. [230] were successful in increasing the lipid metabolism in the diatom *Thalassiosira pseudonana* by targeting knockdown of acyltransferase enzyme locus Thaps3_264297 homologous to human CGI-58, having a conserved histidine-glycine dipeptide, without affecting the growth and viability of *Thalassiosira pseudonana* (whose metabolic network for lipids has been constructed [231]). Using a new dedicated method, Daboussi *et al.* [232] targeted the diatom model organism *Phaeodactylum tricornutum* and used zinc finger nucleases, meganucleases, and transcription activator-like effector nucleases to edit *Phaeodactylum tricornutum* by inducing targeted mutagenesis of certain genes responsible for HVM production. This led to formation of a mutant diatom Tn 19745_1 that produces 45 times more lipid than the control and further increased the HVM production when grown in nutrient-stressed media. However, despite the tremendous reported enhancement, the cost of production of lipids is very high and the process remains uneconomical. In addition, the accumulation of high amounts of HVM inside the cell can prove to be toxic at a level still too low to be relevant for the industry. This is the case for isopentenol, the platform molecule precursor of isoprenoids (for biosynthetic pathways, see [14] for which the lower limit is of the order of 1 g L^−1^ [122]. Therefore, one should see milking as more than an alternative possibility to get more sustainable blue biotech, *i.e.*, as a mandatory process.

Recently, the concept of the cell factory has been expanded to synthetic biology, a branch of life science aiming to develop new biosynthetic pathways for the production of HVM, programmable logic controls to regulate and optimize complex cellular functions, and robust strains. For instance, Harrison and Dunlop [233] have developed a mathematical model for cell growth and biofuel production that involves a synthetic feedback loop using a biosensor to control efflux pump expression. Such a possibility is obviously critical because the cell toxicity of the accumulated HVM can be high (bacteria [234], cyanobacteria [235]). Thus, the export of accumulated HVM out from the producing cells constitutes a major challenge in achieving high-level production. To reach this goal, transporters facilitating the exportation of HVM should be identified at the molecular level and eventually heterologously expressed in the microalgae to be milked. Of course, the resulting protein(s) have to be addressed to the plasma membrane (see [236]). The proof of concept for such a strategy has been obtained by overexpressing several types of transporting systems in the plasma membrane of *Escherichia coli* in order to export isoprenoids [121,122] (Table 4).

To this end, knowledge of diatom physiology has to be deepened to understand the biochemical, cellular and molecular mechanisms sustaining the production of HVM as well as those that may be triggered by milking. This is especially true for carbon metabolism because HVM are mostly made of carbon and hydrogen atoms [236] (see Section 5). *En route* to this goal, the generation of robust mathematical models, able to precisely and accurately describe the metabolic state of a cell will constitute a major advantage, especially if they are able to integrate the different levels of complexity that the production of HVM requires [236,237]. Actually, the CO_2_ concentration and nutrient sources must be investigated and optimized for each microalgal strain [238].

**Table 4:** Cloning of efflux pumps facilitate the excretion of synthesized lipids out from bacteria cells. “Pro/cons” stands for advantages and disadvantages.

Type of Transporter	Origin	Host Cells	Molecules Transported	Pro/Cons	References
Resistance-nodulation-cell division (RND) family	Gram-negative bacteria, similarities with cyanobacteria	*Escherichia coli*	limonene	Pro: increase the excretion of limonene Con: Large tripartite protein complex [239]	[122]
ATP-binding cassette (ABC)	Bacteria	*Escherichia coli*	carotenoids, squalene, botrycoccene	Pro: present in all 5 kingdoms; import or export molecules and ions across cell membranes	[121,240,241]
Formate transporter (focA)	*Escherichia coli*	*Escherichia coli*	formate		[242]

## 5. Understanding of Diatom (Stress) Biology

Being one of the most important world net primary producers, diatoms play an important ecological role [11,243]. From the evolutionary point of view, the history of diatoms is much more complex than for green algae. It is out of the scope of this paper to detail the different roads through which diatoms have evolved. The interested reader is invited to read recent specialized reviews on this topic [244,245]. To be convinced of this complexity, it is enough to mention that diatom evolution has involved several endosymbiotic events, including with a cyanobacterium and a red alga [246] together with a chlamydial invasion [247]. The gene enrichments provided by these events resulted in a unique set of metabolic and regulation networks [248] that certainly helped diatoms to colonize every type of ecological habitat such as freshwater, brackish, marine and hypersaline environments with wide ranges of temperature, pH, and nutrient availability. Some may be considered extremophiles [145,249]. This colonization capacity reflects a very flexible metabolism allowing them to adapt to various environmental constraints [145,250,251,252,253]. Long-term adaptation mechanisms often involve metabolic shifts, consisting in the production of secondary metabolites [53,163]. Because stress conditions such as salinity [254], nutrient deficiency, temperature and high light stress [255] are “interpreted” by the algae as “dangerous” [91], they accumulate high-energy molecules such as carotenoids and/or lipids [13,53,254,256]. For instance, a nitrogen or silicon depletion stress has been shown to increase oil production in diatoms by a factor of 2 or 3 of their average dry weight [257,258,259]. Desiccation can cause algae to create thick mucus sheaths that are often found to contain starch or oil [260]. Diatoms can store energy in the form of chrysolaminarin or as lipids [261], so we will have to learn how to bias production towards oil. It is usually admitted that microalgal division activity competes with HVM accumulation [53,262,263]. Algae with high oil content, such as *Botryococcus*, grow slowly and can only be harvested a few times a week whereas those containing low amounts of oil, such as *Dunaliella*, divide faster and can be harvested daily. For this reason, most industrial applications use algal strains with 20%–40% lipid content [65]. To ensure a high division rate, diatoms can rely on a greater ability to fix CO_2_ than other phytoplanktonic groups [264]. For instance, under fluctuating light intensities, the diatom *Phaeodactylum tricornutum* was nearly twice more efficient than the green alga *Chlorella vulgaris* [60]. This suggests a strong regulation in the fate of photosynthetically fixed carbon [236,265]. This means that any stress impacting negatively on growth should activate HVM production [13,14,53,266]. For instance, lipid production in *Chlorella emersonii* could be as high as 63% DW when grown under nitrogen stress (Table 1). However, conditions allowing growth and neutral lipid accumulation were found recently for the green microalga *Neochloris oleabundans* [267] (*cf.* [230]). In the philosophy of milking, the rate of alga division does not seem very critical because the HVM production will not rely on biomass production but more on the carbon flux through the dedicated pathways, as observed with an *Escherichia coli* strain secreting isoprenoids thanks to the an overexpressed ABC transporter [121].

In the context of lipid milking, the formation and storage of lipids is of course of particular interest. Diatoms, as other microalgae, store lipids into oleosomes [268] (also called spherosomes, lipid droplets, lipid bodies, oil droplets, *etc*.), the amount of which increases under stress conditions (green algae [269]; diatoms [109,256]). In diatoms, lipid synthesis involves the chloroplast and the endoplasmic reticulum (for a review, see [14]). However, as in higher plants [14], there is probably no direct pathway for fatty acids to move from chloroplasts to oleosomes as is usually assumed by many complex interconnected reactions involving the endoplasmic reticulum. A first indication for such a view has been deduced from a proteomic study of isolated oleosomes from the diatom *Fistulifera solaris* JPCC DA0580. Actually, this study has revealed the presence of one specific protein presenting a quinone protein alcohol dehydrogenase-like domain [270]. Using a fluorescent tag, the protein was found to be targeted to the endoplasmic reticulum where it could be involved in the formation of oleosomes [256]. Transportation of oleosomes could occur by exocytosis as observed with the Chlorophyceae alga *Dunaliella salina* [271].

Formation and composition of oleosomes is of course of particular interest in the context of lipid production because they appear to be a vehicle for oozing (see Section 4). Hildebrand *et al.* [90] assumed that the larger the size of a diatom, the larger its lipid droplets. This expectation was not confirmed by our measurements as no correlation was found between biovolume and total lipid droplet volume within a cell (Figure 4B,C) and biovolume category #1 in this study had the largest visible oil contents (Figure 4C). This lack of correlation extends even to individual diatoms in chains, which can be presumed to be clonal (Manoylov, unpublished observations, cf. [109]), *i.e.*, genetically identical, and to have experienced the same microenvironment. Time lapse observations of lipid droplets in unicellular and colonial diatoms might help understand these variations and may be important for elucidating the optimal conditions for oil production, and for recovering oil synthesis after mechanical milking *cf.* [272,273,274,275,276,277,278,279].

## 6. Conclusions and Perspectives

The study of HVM from microalgae is still in its infancy [204]. Microalgae are fast growing, common, and readily available. The commercial exploitation of microalgae to produce HVM is not new but so far is a niche market because these HVM have no crucial importance for human society. For instance, the ambition to replace a significant part, if not all, of the fossil oil consumption by lipids produced by microalgae, not only constitutes a change of paradigm but also a tremendous change in the market, and consequently, in production. To maximize the benefits of this possibility, it is desirable to have environmentally benign production processes. We have reviewed alternative possibilities to reach this goal via milking of diatoms and illustrated them with original data.

At the time of writing, crude oil prices have dipped, perhaps due to economics [280,281], market manipulation [282], or increased production due to fracking [283,284]. In the short term this volatility is hard on biofuel startups [285], whose cost per US gallon presently varies from $9 to $40 [286]. Nevertheless, we anticipate that in the long run biofuels, perhaps obtained via diatom milking, could technologically fit smoothly into our present transportation and energy sectors, maybe at lower cost, while indeed disrupting many current economic and geopolitical arrangements [287]. The ongoing debate over global warming/climate change, including astronomical phenomena [288,289,290], serves as a backdrop, providing breathing room [291] or a sense of urgency [292] or despondency [293], depending on which working hypotheses one adopts for human *versus* cosmic impact. The very act of working on the science of biofuel production puts scientists directly into a position to influence policy [294,295]. Milking approaches to biofuels may alter their economics, increasing their presently low [296] EROI (energy return on investment). One hour of sunshine falling on Earth is equivalent to a year’s worth of human energy consumption, so there is plenty of room for maneuvering, including the prospect of achieving artificial photosynthesis [297,298]. Perhaps we shall someday milk artificial diatoms.

To date, the Chlorophyta algae have been the subjects of most biofuel research. Genera such as *Chlorella*, *Botryococcus*, *Scenedesmus*, *Neochloris*, and *Chlamydomonas* are the typically researched organisms. Research using *Scenedesmus obliquus* has yielded phenomenal increases of oil by manipulating chemical and physical factors. For instance, Mandal and Mallick [299] were able to increase the oil content by 43% (*cf.* [179]). Algal fuels based on diatoms are beginning to show promise [27,90,157,158,176,300,301], which is reasonable, given their contribution to natural oil deposits [59]. Thus diatoms have the ability to produce large amounts of oil. For example, planktonic diatoms are reported to produce lipids in quantities up to 40% dry cell weight [302]. Cultured marine diatoms had 30%–45% dry weight as lipids [303]. The amount of oil in a single diatom is reported as up to 25% of the algal biomass [90] and could perhaps reach 60% of the nonsilica diatom dry mass [27]. For some diatom species, the amount of oil that could be produced has been projected to reach up to 200 times more oil per hectare than soybeans (reviewed in [27]). Diatoms also have the ability to reproduce quickly and can create very large biovolumes. Diatoms can reproduce and double in as little as five hours under laboratory conditions, and in as little as two days in the environment [27]. How fast milked, stationary diatom cultures will replace their oil is as yet untested.

There is no “puzzling underrepresentation of diatoms in the microalgal biofuels arena” [90], as they have been a focus since 1978 [204]. Biodiesel from microalgae or third generation renewable biofuels could meet the demand [55,56] because they are projected to be capable of generating up to 6–200 times more crude oil per surface area than higher plants [27,57,58]. CO_2_ will be available either from the atmosphere or directly from industries. However, our calculations here indicate that there is not enough industrial CO_2_ waste to convert to biofuel to replace fossil energies used for transport.

Unlike crops like rice, wheat, corn, or sugarcane, which when used for ethanol biodiesels compete with food production, algae can be grown without impacting food production [166]. Algae also have other advantages: (a) algae can be cultivated on non-arable land; (b) they can use non-potable water such as municipal/agricultural waste, which also helps to bioremediate the water [20,21,22,23,24,76,165,197,297,304,305,306,307,308]; and (c) they produce a high per-acre yield [189,303,304].

In a recent technico-economic analysis of alga-derived biofuel under both technical and economic uncertainties associated with the development of biorefinery processes, Brownbridge *et al.* [309] concluded that it is unlikely that algal biodiesel as a primary product can be commercially feasible unless other HVM products are also extracted as exemplified by the new mobile algae refinery [310]. However, it is a well-known engineering dictum that it is difficult to maximize more than one function simultaneously without compromise [311]. Therefore, a better approach is to concentrate on optimizing one function only: for instance, milking diatoms or other algae for biofuel production. Microalga HVM come from the cellular process of photosynthesis and carbon metabolism. Many basic questions about microalgae, such as how the complex, interconnecting metabolic and regulation networks work, are as yet still unanswered. Getting this information requires not only genomic and transcriptomic analyses, but also more integrated omics analyses [236].

## Abbreviations

DW: dry weight
EPA: Eicosapentaenoic acid
EROI: energy return on investment
FAE: Fatty Acid Ester-type
FT-IR: Fast-Fourier InfraRed spectroscopy
HVM: high value molecules
LED: light emitting diode
PBR: photobioreactor
PDMS: polydimethylsiloxane
PEF: pulsed electric field
PUFA: PolyUnsaturated Fatty Acids
SE: standard error
UVC: ultraviolet C band
TAG: TriAcylGlycerol

## Supplementary Information

Supplementary Table S1: Diatom taxa reported per site with samples from 10 different locations in Georgia.

Supplementary Data S2: Measurement of lipid droplets.
