# Correction: Vinayak, V., *et al.* Diatom Milking: A Review and New Approaches. *Marine Drugs* 2015, *13*, 2629–2665

**DOI:** 10.3390/md13127063

**Published:** 2015-12-08

**Authors:** Vandana Vinayak, Kalina M. Manoylov, Hélène Gateau, Vincent Blanckaert, Josiane Hérault, Gaëlle Pencréac’h, Justine Marchand, Richard Gordon, Benoît Schoefs

**Affiliations:** 1 Department of Criminology & Forensic Science, School of Applied Sciences, Dr. H.S. Gour University (Central University), Sagar Madhya Pradesh 470003, India; kapilvinayak@gmail.com; 2 Department of Biological & Environmental Sciences, Georgia College and State University, Milledgeville, GA 31061, USA; kalina.manoylov@gcsu.edu; 3 MicroMar, Mer Molécules Santé, IUML—FR 3473 CNRS, University of Le Mans, Faculté des Sciences et Techniques, Avenue Olivier Messiaen, 72085 Le Mans cedex 9, France; lngateau@gmail.com (H.G.); justine.marchand@univ-lemans.fr (J.M.); 4 MicroMar, Mer Molécules Santé, IUML—FR 3473 CNRS, University of Le Mans, IUT de Laval, Rue des Drs Calmette et Guerin, 53020 Laval Cedex 9, France; vincent.blanckaert@univ-lemans.fr; 5 ChimiMar, Mer Molécules Santé, IUML—FR 3473 CNRS, University of Le Mans, IUT de Laval, Rue des Drs Calmette et Guerin, 53020 Laval Cedex 9, France; josiane.herault@univ-lemans.fr (J.H.); gaelle.pencreach@univ-lemans.fr (G.P.); 6 Gulf Specimen Aquarium & Marine Laboratory, Panacea, FL 32346, USA; DickGordonCan@gmail.com; 7 C.S. Mott Center for Human Growth and Development, Department of Obstetrics & Gynecology, Wayne State University, 275 E. Hancock, Detroit, MI 48201, USA

The following two sentences, appearing on p 2642, need to be modified:

“Using a new dedicated method, Daboussi *et al*. [232] targeted the diatom model organism *Phaeodactylum tricornutum* and used zinc finger nucleases, meganucleases, and transcription activator-like effector nucleases to edit *Phaeodactylum tricornutum* by inducing targeted mutagenesis of certain genes responsible for HVM production. This led to formation of a mutant diatom Tn 19745_1 that produces 45 times more lipid than the control and further increased the HVM production when grown in nutrient-stressed media.”

These sentences should read (changes are indicated in bold)

“Using a new dedicated method, Daboussi *et al*. [232] targeted the diatom model organism *Phaeodactylum tricornutum* and used zinc finger nucleases, meganucleases, and transcription activator-like effector nucleases **TALEN** to edit *Phaeodactylum tricornutum* by inducing targeted mutagenesis of certain genes responsible for HVM production. This led to formation of a mutant diatom Tn 19745_1 that produces 45 times more **TAG** than the control and further increased the HVM production when grown in nutrient-stressed media.”

The authors would like to apologize for any inconvenience caused to the readers by these changes.

