# Supplementary material for: Diatom Milking: A Review and New Approaches
Source: Mar Drugs. 2015 Apr 29;13(5):2629–65. doi: 10.3390/md13052629 (PMC4446598; doi:10.3390/md13052629)
Supplement: Supplementary file 1 [file marinedrugs-13-02629-s001.pdf]

Supplementary Information

Table S1. Diatom taxa reported per site with samples from 10 different locations in Georgia. To indicate species richness, unique taxa across all samples are also presented, repeated taxa between locations are not numbered, but presented in alphabetical order within a site. Taxa followed by a number require additional taxonomic evaluation and consideration as new to science. Numbers were carried across sites. The abbreviation spp. indicates a taxon seen in girdle view and without enough evidence for species separation. See Table 3 for identification of sites.

Unique Taxon Number	Taxon	Site
1	*Achnanthidium affine* (Grunow) Czarnecki	8
2	*Achnanthidium deflexum* (Reimer) Kingston	1
	Achnanthidium deflexum (Reimer) Kingston	6
3	*Achnanthidium exiguum* (Grunow) Czarnecki	1
	Achnanthidium exiguum (Grunow) Czarnecki	2
	Achnanthidium exiguum (Grunow) Czarnecki	5
	Achnanthidium exiguum (Grunow) Czarnecki	8
4	*Achnanthidium minutissimum* (Kützing) Czarnecki	1
	Achnanthidium minutissimum (Kützing) Czarnecki	5
	Achnanthidium minutissimum (Kützing) Czarnecki	6
	Achnanthidium minutissimum (Kützing) Czarnecki	7
	Achnanthidium minutissimum (Kützing) Czarnecki	8
	Achnanthidium minutissimum (Kützing) Czarnecki	9
5	*Achnanthidium saprophilum* (Kobayashi et Mayama) Round et Bukht.	5
6	*Achnanthidium* sp. *1*	5
7	*Achnanthidium* sp. *2*	6
8	*Achnanthidium* sp. *3*	2
9	*Achnanthidium* sp. *4*	8
10	*Achnanthidium* sp. *5*	7
11	*Achnanthidium* sp. *6*	10
12	*Amphora copulata* (Kützing) Schoeman et Archibald	5
	Amphora copulata (Kützing) Schoeman et Archibald	7
	Amphora copulata (Kützing) Schoeman et Archibald	8
	Amphora copulata (Kützing) Schoeman et Archibald	9
13	*Amphora pediculus* (Kützing) Grunow	9
14	*Amphora* sp. *1*	10
15	*Amphora* spp.	10
16	*Amphora veneta* Kützing	9
17	*Aulacoseira ambigua* (Grunow) Simonsen	5
	Aulacoseira ambigua (Grunow) Simonsen	7
	Aulacoseira ambigua (Grunow) Simonsen	8
	Aulacoseira ambigua (Grunow) Simonsen	9
18	*Aulacoseira granulata* var. *angustissima* (Müller) Simonsen	9
	Aulacoseira granulata var. *angustissima* (Müller) Simonsen	8
18	*Aulacoseira italica* (Ehrenberg) Simonsen	7
20	*Aulacoseira* sp. *1*	2

Table S1. *Cont.*

21	*Aulacoseira* sp. *2*	8
22	*Aulacoseira* sp. *3*	7
23	*Aulacoseira* sp. *4*	10
24	*Aulacoseira* spp.	6
25	*Bacillaria paradoxa* Gmelin	2
	Bacillaria paradoxa Gmelin	7
26	*Biddulphia* sp. *1*	10
27	*Biddulphia* sp. *2*	10
28	*Biremis circumtexta* (Meister ex Hustedt) Lange-Bertalot et Witk.	10
29	*Caloneis bacillum* (Grunow) Cleve	5
	Caloneis bacillum (Grunow) Cleve	7
	Caloneis bacillum (Grunow) Cleve	8
30	*Caloneis* sp. *1*	2
31	*Campylosira cymbelliformis* (Schmidt) Grunow in Van Heurck	10
32	*Cocconeis disculus* (Schumann) Cleve	10
33	*Cocconeis neodiminuta* Krammer	10
34	*Cocconeis pediculus* Ehrenberg	9
35	*Cocconeis placentula* Ehrenberg	4
	Cocconeis placentula Ehrenberg	10
36	*Cocconeis placentula* var. *euglypta* (Ehrenberg) Grunow	4
37	*Cocconeis placentula* var. *lineata* (Ehrenberg) Van Heurck	6
38	*Coscinodiscus* sp. *1*	10
39	*Craticula ambigua* (Ehrenberg) Mann in Round, Crawford and Mann	5
	Craticula ambigua (Ehrenberg) Mann in Round, Crawford and Mann	6
40	*Craticula cuspidata* (Kützing) Mann	5
	Craticula cuspidata (Kützing) Mann	9
41	*Craticula halophila* (Grunow ex Van Heurck) Mann	6
	Craticula halophila (Grunow ex Van Heurck) Mann	10
42	*Craticula molestiformis* (Hustedt) Mayama	5
	Craticula molestiformis (Hustedt) Mayama	8
43	*Cyclostephanos invisitatus* (Hohn et Hellerman) Theriot, Stoermer et Håk.	6
	Cyclostephanos invisitatus (Hohn et Hellerman) Theriot, Stoermer et Håk.	1
44	*Cyclotella atomus* Hustedt	8
	Cyclotella atomus Hustedt	2
	Cyclotella atomus Hustedt	10
45	*Cyclotella litoralis* Lange & Syvertsen	10
46	*Cyclotella meneghiniana* Kützing	6
	Cyclotella meneghiniana Kützing	1
47	*Cyclotella* sp. *2*	10
48	*Cylindrotheca gracilis* (Brébisson) Grunow	10
49	*Cymatopleura solea* var. *apiculata* (Smith) Ralfs	2
50	*Cymatosira belgica* Grunow	10
51	*Cymatosira Lorenziana* Grunow	10
52	*Delphineis surirella* (Ehrenberg) Andrews	10
53	*Denticula kuetzingii* Grunow	6

Table S1. *Cont.*

54	*Diadesmis confervacea* Kützing	1
	Diadesmis confervacea Kützing	5
55	*Diadesmis contenta* (Grunow ex Van Heurck) Mann	5
	Diadesmis contenta (Grunow ex Van Heurck) Mann	8
56	*Diploneis aestuari* Hustedt	10
57	*Diploneis pseudobombiformis* Hustedt	10
58	*Discostella pseudostelligera* (Hustedt) Houk et Klee	10
59	*Discostella stelligera* (Cleve et Grunow in Cleve) Houk et Klee	2
	Discostella stelligera (Cleve et Grunow in Cleve) Houk et Klee	5
	Discostella stelligera (Cleve et Grunow in Cleve) Houk et Klee	6
	Discostella stelligera (Cleve et Grunow in Cleve) Houk et Klee	8
60	*Encyonema silesiacum* (Bleisch) Mann	8
61	*Encyonema triangulum* (Ehrenberg) Kützing	6
62	*Entomoneis alata* (Ehrenberg) Ehrenberg	6
	Entomoneis alata (Ehrenberg) Ehrenberg	10
63	*Eolimna minima* (Grunow) Lange-Bertalot	1
	Eolimna minima (Grunow) Lange-Bertalot	5
	Eolimna minima (Grunow) Lange-Bertalot	7
	Eolimna minima (Grunow) Lange-Bertalot	9
64	*Eolimna tantula* (Hustedt) Lange-Bertalot in Werum and Lange-Bertalot	5
65	*Epithemia adnata* (Kützing) Brébisson	5
66	*Epithemia turgida* (Ehrenberg) Kützing	1
67	*Eunotia bilunaris* (Ehrenberg) Mills	5
	Eunotia bilunaris (Ehrenberg) Mills	7
	*Eunotia bilunari*s (Ehrenberg) Souza	2
68	*Eunotia meisteri* Hustedt	8
69	*Eunotia pectinali*s (Müller) Rabenhorst	2
	*Eunotia pectinali*s (Müller) Rabenhorst	5
	*Eunotia pectinali*s (Müller) Rabenhorst	6
	*Eunotia pectinali*s (Müller) Rabenhorst	7
70	*Eunotia pectinalis* var. *undulata* (Ralfs) Rabenhorst	8
71	*Eunotia soleirolii* (Kützing) Rabenhorst	5
	Eunotia soleirolii (Kützing) Rabenhorst	8
72	*Eunotia* sp. *1*	5
	Eunotia sp. *1*	8
73	*Eunotia* sp. *2*	5
	Eunotia sp. *2*	8
74	*Eunotia* sp. *3*	6
75	*Eunotia* sp. *4*	1
76	*Eunotia* sp. *5*	8
77	*Eunotia* sp. *6*	5
78	*Fragilaria capucina* Desmazières	7
	Fragilaria capucina Desmazières	5
79	*Fragilaria gracilis* Østrup	5
80	*Fragilaria mesolepta* Rabenhorst	6
81	*Fragilaria parasitica* (Smith) Grunow in Van Heurck	5

Table S1. *Cont.*

82	*Fragilaria sepes* Ehrenberg	8
83	*Fragilaria* sp. *1*	8
84	*Fragilaria tenera* (Smith) Lange-Bertalot	7
85	*Frustulia crassinervia* (Brébisson) Lange-Bertalot et Krammer	8
	Frustulia crassinervia (Brébisson) Lange-Bertalot et Krammer	7
86	*Frustulia vulgaris* (Thwaites) De Toni	8
	Frustulia vulgaris (Thwaites) De Toni	5
87	*Gomphonema acidoclinatum* Lange-Bertalot et Reichardt	5
88	*Gomphonema affine* Kützing	6
	Gomphonema affine Kützing	2
89	*Gomphonema angustum* Agardh	7
90	*Gomphonema gracile* Ehrenberg	6
	Gomphonema gracile Ehrenberg	2
	Gomphonema gracile Ehrenberg	5
91	*Gomphonema hebridense* Gregory	5
92	*Gomphonema innocens* Reichardt	5
93	*Gomphonema lippertii* E.Reichardt et Lange-Bertalot	7
	Gomphonema lippertii E.Reichardt et Lange-Bertalot	5
94	*Gomphonema minutum* (Agardh) Agardh	1
95	*Gomphonema olivaceoides* Hustedt	6
96	*Gomphonema parvulum* (Kützing) Kützing	1
	Gomphonema parvulum (Kützing) Kützing	5
	Gomphonema parvulum (Kützing) Kützing	6
	Gomphonema parvulum (Kützing) Kützing	7
	Gomphonema parvulum (Kützing) Kützing	8
97	*Gomphonema pseudobohemicum* Lange-Bertalot and Reichardt	5
98	*Gomphonema pumilum* (Grunow) Reichardt et Lange-Bertalot	8
99	*Gomphonema* sp. *1*	8
	Gomphonema sp. *1*	1
	Gomphonema sp. *1*	5
	Gomphonema sp. *1*	6
	Gomphonema sp. *1*	7
100	*Gomphonema* sp. *2*	1
101	*Gomphonema* sp. *3*	5
102	*Gomphonema* sp. *4*	6
103	*Gomphonema* sp. *5*	7
104	*Gomphonema* spp.	8
	Gomphonema spp.	1
105	*Gyrosigma kuetzingii* (Grunow) Cleve	5
106	*Gyrosigma* sp. *1*	10
107	*Gyrosigma* sp. *2*	10
108	*Gyrosigma* sp. *3*	10
109	*Gyrosigma* sp. *4*	10
110	*Gyrosigma* sp. *5*	10
111	*Gyrosigma* sp. *6*	10

Table S1. *Cont.*

112	*Gyrosigma* sp. *7*	10
113	*Gyrosigma* sp. *8*	10
114	*Gyrosigma* sp. *9*	10
115	*Gyrosigma* sp. *10*	10
116	*Gyrosigma* sp. *11*	10
117	*Halamphora salinicola* Levkov et Díaz	10
118	*Handmannia comta* (Ehrenberg) Kociolek & Khursevich	10
119	*Hippodonta capitata* (Ehrenberg) Lange-Bertalot, Metzeltin et Witkowski	8
	Hippodonta capitata (Ehrenberg) Lange-Bertalot, Metzeltin et Witkowski	1
	Hippodonta capitata (Ehrenberg) Lange-Bertalot, Metzeltin et Witkowski	6
120	*Hippodonta hungarica* (Grunow) Lange-Bertalot, Metzeltin et Witkowski	6
121	*Karayevia clevei* (Grunow) Bukhtiyarova	5
122	*Luticola mutica* (Kützing) Mann	5
	Luticola mutica (Kützing) Mann	5
123	*Mayamaea permitis* (Hustedt) Bruder and Medlin	1
124	*Melosira varians* Agardh	6
125	*Meridion circulare* var. *constrictum* (Ralfs) Van Heurck	5
	Meridion circulare var. *constrictum* (Ralfs) Van Heurck	7
126	*Minidiscus* sp. *1*	10
127	*Navicula angusta* Grunow	6
128	*Navicula bicephala* Hustedt	8
	Navicula bicephala Hustedt	7
129	*Navicula canalis* Patrick	1
130	*Navicula cryptocephala* Kützing	1
	Navicula cryptocephala Kützing	2
	Navicula cryptocephala Kützing	5
	Navicula cryptocephala Kützing	6
	Navicula cryptocephala Kützing	7
	Navicula cryptocephala Kützing	8
	Navicula cryptocephala Kützing	9
131	*Navicula cryptotenella* Lange-Bertalot	1
	Navicula cryptotenella Lange-Bertalot	4
	Navicula cryptotenella Lange-Bertalot	5
132	*Navicula difficillima* Hustedt	5
	Navicula difficillima Hustedt	8
133	*Navicula erifuga* Lange-Bertalot	6
	Navicula erifuga Lange-Bertalot	10
134	*Navicula gregaria* Donkin	9
135	*Navicula halinae* Witkowski	10
136	*Navicula indifferens* Hustedt	1
137	*Navicula ingenua* Hustedt	1
138	*Navicula notha* Wallace	1
139	*Navicula pavillardi* Hustedt	10
140	*Navicula recens* (Lange-Bertalot) Lange-Bertalot	2
	Navicula recens (Lange-Bertalot) Lange-Bertalot	10
141	*Navicula reichardtiana* Lange-Bertalot	4

Table S1. *Cont.*

142	*Navicula salinicola* Hustedt	10
143	*Navicula schadei* Krasske	5
	Navicula schadei Krasske	8
144	*Navicula* sp. *1*	2
145	*Navicula* sp. *2*	8
146	*Navicula* sp. *3*	7
147	*Navicula* sp. *4*	10
148	*Navicula* sp. *5*	6
149	*Navicula* sp. *6*	9
150	*Navicula* sp. *7*	2
151	*Navicula* sp. *8*	6
	Navicula sp. *8*	9
	Navicula sp. *8*	10
152	*Navicula* sp. *9*	6
153	*Navicula* sp. *10*	6
154	*Navicula* sp. *11*	6
155	*Navicula* sp. *12*	6
156	*Navicula* sp. *13*	6
157	*Navicula* sp. *14*	6
158	*Navicula* sp. *15*	6
159	*Navicula* sp. *16*	6
160	*Navicula* sp. *17*	6
161	*Navicula* sp. *18*	6
162	*Navicula* sp. *19*	6
163	*Navicula subminuscula* Manguin	2
164	*Navicula tenelloides* Hustedt	8
165	*Navicula tripunctata* (Müller) Bory	5
166	*Navicula trivialis* Lange-Bertalot	1
167	*Navicula veneta* Kützing	1
	Navicula veneta Kützing	4
168	*Navicula viridula* (Kützing) Kützing	5
169	*Neidium ampliatum* (Ehrenberg) Krammer	8
170	*Neidium dubium* (Ehrenberg) Cleve	5
171	*Neidium* sp. *1*	5
172	*Nitzschia acicularis* (Kützing) Smith	2
	Nitzschia acicularis (Kützing) Smith	5
173	*Nitzschia agnita* Hustedt	4
	Nitzschia agnita Hustedt	10
174	*Nitzschia amphibia* Grunow	6
	Nitzschia amphibia Grunow	2
	Nitzschia amphibia Grunow	5
175	*Nitzschia amphibioides* Hustedt	3
176	*Nitzschia angustata* (Smith) Grunow	2
177	*Nitzschia archibaldii* Lange-Bertalot	2
178	*Nitzschia compressa* (Bailey) Boyer	6
179	*Nitzschia desertorum* Hustedt	2

Table S1. *Cont.*

180	*Nitzschia dissipata* (Kützing) Grunow	10
181	*Nitzschia dubia* Smith	6
182	*Nitzschia filiformis* (Smith) Van Heurck	1
183	*Nitzschia fonticola* (Grunow) Grunow	9
	Nitzschia fonticola (Grunow) Grunow	1
184	*Nitzschia fossilis* (Grunow) Grunow	1
185	*Nitzschia frustulum* (Kützing) Grunow	2
	Nitzschia frustulum (Kützing) Grunow	7
186	*Nitzschia gracilis* Hantzsch	1
187	*Nitzschia granulata* Grunow	10
188	*Nitzschia inconspicua* Grunow	1
	Nitzschia inconspicua Grunow	4
189	*Nitzschia lacuum* Lange-Bertalot	6
190	*Nitzschia laevis* Hustedt	10
191	*Nitzschia lanceola* Grunow	10
192	*Nitzschia lanceolata* Smith	10
193	*Nitzschia liebethruthii* Rabenhorst	2
	Nitzschia liebethruthii Rabenhorst	4
194	*Nitzschia lorenziana* Grunow	10
195	*Nitzschia microcephala* Grunow	1
196	*Nitzschia nana* Grunow ex Van Heurck	2
197	*Nitzschia palea* (Kützing) Smith	1
	Nitzschia palea (Kützing) Smith	4
	Nitzschia palea (Kützing) Smith	8
198	*Nitzschia palea* var. *debilis* (Kützing) Grunow	2
	Nitzschia palea var. *debilis* (Kützing) Grunow	4
	Nitzschia palea var. *debilis* (Kützing) Grunow	5
	Nitzschia palea var. *debilis* (Kützing) Grunow	6
	Nitzschia palea var. *debilis* (Kützing) Grunow	7
	Nitzschia palea var. *debilis* (Kützing) Grunow	8
199	*Nitzschia palea* var. *tenuirostris* Grunow	8
200	*Nitzschia paleacea* Grunow	1
201	*Nitzschia paleaeformis* Hustedt	5
203	*Nitzschia sigma* (Kützing) Smith	10
204	*Nitzschia* sp. *1*	6
	Nitzschia sp. *1*	8
205	*Nitzschia* sp. *2*	1
206	*Nitzschia* sp. *3*	6
207	*Nitzschia* sp. *4*	4
208	*Nitzschia* sp. *5*	8
209	*Nitzschia* sp. *6*	5
210	*Nitzschia* sp. *9*	9
211	*Nitzschia* sp. *10*	5
212	*Nitzschia* sp. *11*	8
213	*Nitzschia* sp. *12*	10
214	*Nitzschia* sp. *13*	2

Table S1. *Cont.*

215	*Nitzschia* sp. *14*	6
216	*Nitzschia* spp.	2
217	*Nitzschia subacicularis* Hustedt	4
218	*Nupela* sp. *5*	8
219	*Paralia sulcata* (Ehrenberg) Cleve	10
220	*Pinnularia borealis* var. *rectangularis* Carlson	8
221	*Pinnularia gibba* (Ehrenberg) Ehrenberg	2
222	*Pinnularia legumen* Ehrenberg	8
	Pinnularia legumen Ehrenberg	5
223	*Pinnularia obscura* Krasske	8
	Pinnularia obscura Krasske	5
224	*Pinnularia schroederii* (Hustedt) Krammer	5
225	*Pinnularia* sp. *1*	2
	Pinnularia sp. *1*	10
226	*Pinnularia* sp. *2*	9
227	*Pinnularia* sp. *3*	5
228	*Pinnularia* sp. *4*	8
229	*Pinnularia* sp. *5*	10
230	*Pinnularia* sp. *6*	2
231	*Pinnularia* sp. *7*	6
232	*Pinnularia spp*	6
233	*Pinnularia subcapitata* Gregory	1
	Pinnularia subcapitata Gregory	5
	Pinnularia subcapitata Gregory	7
234	*Pinnularia viridiformis* var. *minor* Krammer	5
235	*Pinnularia viridis* (Nitzsch) Ehrenberg	5
236	*Placoneis symmetrica* (Hustedt) Lange-Bertalot	5
237	*Planothidium delicatulum* (Kützing) Round et Bukhtiyarova	6
238	*Planothidium dubium* (Grunow) Round et Bukhtiyarova	9
	Planothidium dubium (Grunow) Round et Bukhtiyarova	1
239	*Planothidium frequentissimum* (Lange-Bertalot) Lange-Bertalot	6
	Planothidium frequentissimum (Lange-Bertalot) Lange-Bertalot	2
	Planothidium frequentissimum (Lange-Bertalot) Lange-Bertalot	5
240	*Planothidium lanceolatum* (Brébisson ex Kützing) Lange-Bertalot	2
	Planothidium lanceolatum (Brébisson ex Kützing) Lange-Bertalot	4
	Planothidium lanceolatum (Brébisson ex Kützing) Lange-Bertalot	7
241	*Planothidium rostratum* (Østrup) Lange-Bertalot	8
	Planothidium rostratum (Østrup) Lange-Bertalot	5
	Planothidium rostratum (Østrup) Lange-Bertalot	7
242	*Pleurosigma angulatum* (Quekett) Smith	5
243	*Pleurosigma* sp. *1*	10
244	*Pleurosigma* sp. *2*	10
245	*Pleurosigma* sp. *3*	10
246	*Pleurosigma* sp. *4*	10
247	*Pleurosigma* sp. *5*	10
248	*Psammothidium ventralis* (Krasske) Bukhtiyarova and Round	7

Table S1. *Cont.*

249	*Rhaphoneis amphiceros* (Ehrenberg) Ehrenberg	10
250	*Rhaphoneis grossepunctata* Hustedt	10
251	*Rhizosolenia* sp. *1*	10
252	*Rhoicosphenia abbreviata* (Agardh) Lange-Bertalot	1
	Rhoicosphenia abbreviata (Agardh) Lange-Bertalot	4
253	*Sellaphora laevissima* (Kützing) Mann	5
254	*Sellaphora parapupula* Lange-Bertalot	5
255	*Sellaphora pupula* (Kützing) Meresckowsky	8
	Sellaphora pupula (Kützing) Meresckowsky	5
	Sellaphora pupula (Kützing) Meresckowsky	6
	Sellaphora pupula (Kützing) Meresckowsky	7
	Sellaphora pupula (Kützing) Meresckowsky	9
256	*Sellaphora rectangularis* (Gregory) Lange-Bertalot and Metzeltin	7
257	*Sellaphora seminulum* (Grunow) Mann	1
258	*Skeletonema costatum* (Greville) Cleve	10
259	*Stauroneis anceps* Ehrenberg	5
260	*Stauroneis gracilior* Reichardt	5
261	*Stauroneis nobilis* Schumann	5
262	*Stauroneis phoenicenteron* (Nitzsch) Ehrenberg	5
263	*Stauroneis smithii* var. *incisa* Pantocsek	7
264	*Staurosira construens* Ehrenberg	8
	Staurosira construens Ehrenberg	5
	Staurosira construens Ehrenberg	7
265	*Staurosira construens* var. *venter* (Ehrenberg) Hamilton	1
266	*Staurosira* sp. *1*	9
267	*Staurosira* sp. *2*	5
268	*Staurosira* sp. *3*	8
269	*Staurosira* sp. *4*	10
270	*Staurosira* sp. *5*	2
271	*Staurosira* sp. *6*	6
272	*Staurosirella pinnata* (Ehrenberg) Williams et Round	8
	Staurosirella pinnata (Ehrenberg) Williams et Round	5
	Staurosirella pinnata (Ehrenberg) Williams et Round	7
	Staurosirella pinnata (Ehrenberg) Williams et Round	9
	Staurosirella pinnata (Ehrenberg) Williams et Round	10
273	*Surirella angusta* Kützing	1
	Surirella angusta Kützing	5
	Surirella angusta Kützing	9
274	*Surirella* sp. *1*	5
	Surirella sp. *1*	7
275	*Surirella* sp. *2*	9
276	*Surirella* sp. *3*	5
277	*Surirella* sp. *4*	8
278	*Surirella* sp. *5*	9
279	*Surirella* sp. *6*	2
280	*Surirella* sp. *7*	6

Table S1. *Cont.*

281	*Surirella* sp. *8*	5
282	*Surirella* sp. *9*	10
283	*Synedra* sp. *1*	5
284	*Synedra* sp. *2*	2
	Synedra sp. *2*	8
	Synedra sp. *2*	9
285	*Synedra* sp. *3*	5
	Synedra sp. *3*	6
	Synedra sp. *3*	10
286	*Tabellaria flocculosa* (Roth) Kützing	9
287	*Terpsinoë musica* Ehrenberg	10
288	*Thalassionema pseudonitzschioide*s (Schuette et Schrader) Hasle	10
289	T*halassionema* sp. *6*	10
290	*Thalassiosira pseudonana* Hasle et Heimdal	1
291	*Thalassiosira* sp. *1*	10
292	*Thalassiosira* sp. *2*	8
	Thalassiosira sp. *2*	10
293	*Thalassiosira* sp. *3*	10
294	*Thalassiosira* sp. *4*	2
295	*Thalassiosira* sp. *5*	6
296	*Thalassiosira* sp. *6*	5
	Thalassiosira sp. *6*	9
297	*Thalassiosira weissflogii* (Grunow) Fryxell et Hasle	2
298	*Tryblionella apiculata* Gregory	6
299	*Tryblionella levidensis* Smith	8
300	*Tryblionella* sp. *1*	10
301	*Tryblionella* sp. *2*	6
302	*Tryblionella* sp. *3*	2
303	*Tryblionella* sp. *4*	8
304	*Tryblionella* sp. *5*	7
305	*Tryblionella* sp. *6*	2
306	*Tryblionella* sp. *7*	2
307	*Tryblionella* sp. *8*	10
308	*Tryblionella* sp. *9*	10
309	*Tryblionella* sp. *10*	10
310	*Tryblionella* sp. *11*	10
311	*Tryblionella* sp. *12*	10
312	*Tryblionella* sp. *13*	10
313	*Tryblionella* sp. *14*	10
314	*Tryblionella* sp. *15*	10
315	*Tryblionella* sp. *16*	10
316	*Tryblionella* sp. *17*	10
317	*Tryblionella* sp. *18*	10
318	*Tryblionella* sp. *19*	10
319	*Tryblionella* sp. *20*	10
320	*Tryblionella* sp. *21*	10

Table S1. *Cont.*

321	*Tryblionella* sp. *22*	10
322	*Tryblionella* sp. *23*	10
323	*Tryblionella* sp. *24*	10
324	*Tryblionella* sp. *25*	10
325	*Tryblionella* sp. *26*	10
326	*Tryblionella* sp. *27*	10
327	*Tryblionella* sp. *28*	10
328	*Tryblionella* sp. *29*	10
329	*Ulnaria acus* (Kützing) Aboal	5
330	*Ulnaria delicatissima* (W. Smith) Aboal et Silva	8
331	*Ulnaria delicatissima* var. *angustissima* (Grunow) Aboal	7
332	*Ulnaria ulna* (Nitzsch) Compère	10
333	Undetermined Centric sp. 1	7
334	Undetermined Pennate	10
335	Undetermined Pennate sp. 1	5
	Undetermined Pennate sp. 1	8
336	Undetermined Pennate sp. 2	5

Supplementary Data S2

Measurement of oil droplets: Diatoms used in Figure 3 were collected in Georgia (USA) (see Table 1). All observations were performed with a 40× objective lens and 10× eyepiece on a Lumar scope or Leica DM2500 microscope equipped with differential interference contrast (DIC). Under 400× magnification most diatoms were identified to genus. Images were captured and specimens were measured using a 3 megapixel Leica DFC295 color digital camera. The following morphological characters were recorded: length of apical axis; maximal length of transapical axis at widest part, chloroplast shape and number. Biovolume was calculated by approximating the shape of the diatoms to a geometric shape [1]. Oil droplets were approximated to spheres and the total volume of the spheres was calculated by measuring the diameter of each visible oil droplet and multiplying the number of spheres of respective size added together. The fine focus was traversed up and down to ensure observation of all visible oil droplets within the cells and the maximum diameter of each oil droplet in a through focal scan was obtained. Diatoms were classified in 5 categories based on biovolume: (1) representatives of *Cymatoseira*, *Eunotia*, *Achnanthidium* and *Cyclotella* 10–500 μm^3; (2) *Fragilaria*, some *Navicula* and *Nitzschia* species 501–1000 μm^3; (3) *Craticula*, 1001–1500 μm^3; (4) Representatives of *Synedra*, *Nitzschia*, *Gomphoneis*, *Gyrosigma*, *Pleurosigma*, large *Navicula* species: 1501–4000 μm^3; (5) *Pleurosira*, *Synedra* and *Terpsinoë* >5000 μm^3. Categories are based on the fact that most diatoms from these environments in Southeastern Georgia have a biovolume less than 3000 μm^3. Our approach of classifying cell biovolume of the very diverse diatom community into only 5 categories accounted for the averages of total oil droplet volume within category #1 appearing to be larger than the average cell biovolume. This is an artifact of the 50-fold range of diatom volumes in category #1.

Accumulation of lipid as oil droplets has been reported as visible under light microscopy (reviewed in: [2–4]). Thus, for this project, only presence/absence and size of obvious, refractile oil droplets were

documented, comparable to those portrayed elsewhere [4–6]. Oil droplets could have been slightly overestimated in size due to diffraction effects. Only opaque, refractile, colored spheres within the cells were considered to be oil droplets, but it is possible that some granules or other cellular material were included, and that nonspherical oil droplets were excluded. Although it was suggested that "…Stoermer & Pankratz [7] report extremely large oil bodies, occupying the entire vacuole in *Amphipleura pellucida*…" [3] (cf. Figure 3 in [8]), there is no discussion of such in that paper [7] or its sequel, in which it is stated instead: "Large vacuoles fill most of the portions of the cell distal to the central cytoplasmic bridge. These are crossed by cytoplasmic strands which often contain oil bodies of varying sizes" [9].

We did not attempt to locate the oil droplets we observed relative to the vacuoles. (Diatoms have one or two vacuoles, which are clear [10], sometimes many [11].) Oil droplets can occur inside [9,12] or outside vacuoles [10,11,13]. Of course, we could not see the initial, visually submicroscopic oil droplets that form near [14] or within [15] the pyrenoids, as shown by electron microscopy. Thus visual microscopy may alternatively underestimate the total oil present. For future work, vital lipid stains [16], vital fluorescent dyes [6,17,18] for lipids, or noninvasive color/spectral difference image processing [19,20] might be useful for quantitating how much oil can be milked and the production and recovery of production of oil in single diatoms.

References

1. Hillebrand, H.; Durselen, C.D.; Kirschtel, D.; Pollingher, U.; Zohary, T. Biovolume calculation for pelagic and benthic microalgae. *J. Phycol.* **1999**, *35*, 403–424.
2. Smith, G.M. *The Fresh-Water Algae of the United States*, 2nd ed.; McGraw-Hill Book Co.: New York, NY, USA, 1950.
3. Duke, E.L.; Reimann, B.E.F. The ultrastructure of the diatom cell. In *The Biology of Diatoms*; Werner, D., Ed.; Blackwell Scientific Publications: Oxford, UK, 1977; pp. 65–109.
4. Lauritis, J.A.; Coombs, J.; Volcani, B.E. Studies on the biochemistry and fine structure of silica shell formation in diatoms IV. Fine structure of the apochlorotic diatom *Nitzschia alba* Lewin and Lewin. *Arch. Mikrobiol.* **1968**, *62*, 1–16.
5. Ramachandra, T.V.; Mahapatra, D.M.; Karthick B.; Gordon, R. Milking diatoms for sustainable energy: biochemical engineering versus gasoline-secreting diatom solar panels. *Ind. Eng. Chem. Res.* **2009**, *48*, 8769–8788.
6. Hildebrand, M.; Davis, A.K.; Smith, S.R.; Traller, J.C.; Abbriano, R. The place of diatoms in the biofuels industry. *Biofuels* **2012**, *3*, 221–240.
7. Stoermer, E.F.; Pankratz, H.S. Fine structure of diatom *Amphipleura pellucida*. I. Wall structure. *Am. J. Bot.* **1964**, *51*, 986–990.
8. Drum, R.W.; Pankratz, H.S. Post mitotic fine structure of *Gomphonema parvulum*. *J. Ultrastruct. Res.* **1964**, *10*, 217–223.
9. Stoermer, E.F.; Pankratz, H.S.; Bowen, C.C. Fine structure of the diatom *Amphipleura pellucida*. II. Cytoplasmic fine structure and frustule formation. *Am. J. Bot.* **1965**, *52*, 1067–1078.
10. Round, F.E.; Crawford, R.M.; Mann, D.G. *The Diatoms, Biology & Morphology of the Genera*; Cambridge University Press: Cambridge, UK, 1990.

11. Anderson, O.R. The ultrastructure and cytochemistry of resting cell formation in *Amphora coffaeformis* (Bacillariophyceae). *J. Phycol.* **1975**, *11*, 272–281.
12. Yang, S.; Wu, R.S.S.; Kong, R.Y.C. Physiological and cytological responses of the marine diatom *Skeletonema costatum* to 2,4-dichlorophenol. *Aquat. Toxicol.* **2002**, *60*, 33–41.
13. Crawford, R.M. The protoplasmic ultrastructure of the vegetative cell of *Melosira varians* C.A. Agardh. *J. Phycol.* **1973**, *9*, 50–61.
14. Drum, R.W.; Pankratz, H.S. Pyrenoids, raphes and other fine structure in diatoms. *Am. J. Bot.* **1964**, *51*, 405–418.
15. Stoermer, E.F.; Pankratz, H.S.; Drum, R.W. The fine structure of *Mastogloia grevillei* Wm. Smith. *Protoplasma* **1964**, *59*, 1–13.
16. Enghusen, E.; Enghusen, K. Einige Untersuchungen über Vitalfärbung und Vitalfarbstoffe. *Acta Anat.* **1961**, *45*, 177–201.
17. Cooper, M.S.; Hardin, W.R.; Petersen, T.W.; Cattolico, R.A. Visualizing "green oil" in live algal cells. *J. Biosci. Bioeng.* **2010**, *109*, 198–201.
18. Govender, T.; Ramanna, L.; Rawat, I.; Bux, F. BODIPY staining, an alternative to the Nile Red fluorescence method for the evaluation of intracellular lipids in microalgae. *Bioresour. Technol.* **2012**, *114*, 507–511.
19. Weeks, A.R., Jr. *Fundamentals of Electronic Image Processing*; SPIE Optical Engineering Press: Bellingham, WA, USA, 1996.
20. Holmes, T.; Larson, J.; Turturro, M.; Vaicik, M.; Papavasiliou, G.; Larkin, S.; Holmes, C.D.; Jurkevich, A.; Sinha, S.; Toshihiko, E.; *et al*. Multimodality, multispectral and 3D light microscopy of engineered tissues without dyes. *J. Tissue Eng. Regen. Med.* **2012**, *6*, 335.

© 2015 by the authors; licensee MDPI, Basel, Switzerland. This article is an open access article distributed under the terms and conditions of the Creative Commons Attribution license (http://creativecommons.org/licenses/by/4.0/).
